# X-ray data from a cyclic tensile study of melt-spun poly(3-hydroxybutyrate) P3HB fibers: A reversible mesophase

**DOI:** 10.1016/j.dib.2019.104376

**Published:** 2019-08-12

**Authors:** Edith Perret, Felix A. Reifler, Ali Gooneie, Rudolf Hufenus

**Affiliations:** a Laboratory for Advanced Fibers, Empa, Swiss Federal Laboratories for Materials Science and Technology, Lerchenfeldstrasse 5, 9014, St. Gallen, Switzerland; b Center for X-ray Analytics, Empa, Swiss Federal Laboratories for Materials Science and Technology, Überlandstrasse 129, 8600, Dübendorf, Switzerland

**Keywords:** Wide-angle x-ray diffraction, Small-angle x-ray scattering, P3HB, poly(3-hydroxybutyrate), Biodegradable, Melt-spun, Molecular dynamics simulations

## Abstract

Wide-angle x-ray diffraction (WAXD) patterns that show mesophases in core-sheath bicomponent fibers and amorphous fibers are presented in section 1.1 of the article. Section 1.2 presents molecular dynamics simulations and scattered intensity calculations of stretched P3HB chains. Sections 1.3–1.6 summarize WAXD and small-angle x-ray scattering (SAXS) data analysis from a tensile study of melt-spun P3HB fibers. Azimuthal profiles are extracted from 2D WAXD patterns at various angular regions and the positions of equatorial reflections and corresponding d-spacings are summarized. Additionally, the extracted structural parameters from SAXS images are summarized. The tensile stress calculations, crystal orientation calculations, applied intensity corrections, calculations of long spacings, coherence lengths and lamellar diameters are explained in the methods subsections 2.3.1–2.3.7. WAXD and SAXS measurements of P3HB fibers were recorded on a Bruker Nanostar U diffractometer (Bruker AXS, Karlsruhe, Germany). The recorded WAXD/SAXS patterns were analyzed with the evaluation software DIFFRAC.EVA (version 4.2., Bruker AXS, Karlsruhe, Germany) and python codes. For more information see ‘Tensile study of melt-spun poly(3-hydroxybutyrate) P3HB fibers: Reversible transformation of a highly oriented phase’ (Perret et al., 2019).

**Table:** Specifications Table

Subject	Materials Science: Polymers and Plastics
Specific subject area	Biodegradable melt-spun monofilaments.
Type of data	Table Image Figure Equations
How data were acquired	Instruments: Xcalibur PX four-circle single crystal diffractometer Bruker Nanostar U diffractometer Software: DIFFRAC.EVA (version 4.2., Bruker AXS, Karlsruhe, Germany) Python codes
Data format	Raw Analyzed
Parameters for data collection	Wide-angle x-ray diffraction and small-angle x-ray scattering patterns were taken in-situ during tensile loading of P3HB fibers.
Description of data collection	WAXD and SAXS patterns of a P3HB monofilament were recorded on a Bruker Nanostar U diffractometer (Bruker AXS, Karlsruhe, Germany) with a beam defining pinhole of 300 μm, with Cu Kα radiation (λ = 1.5419 Å) and a VÅNTEC-2000 MikroGap area detector. The tensile stress was applied using a TS 600 tensile stage (Anton Paar GmbH, Graz, Austria) equipped with load cell LC-5N. Single filaments were used for all WAXD and SAXS measurements, which were performed in two separate experiments with distances of 16.8 cm and 144.4 cm, respectively, between the sample and the active detector area.
Data source location	Empa, St. Gallen, Switzerland
Data accessibility	Mendeley Data DOI: 10.17632/h62v8nv8ck.1 https://doi.org/10.17632/h62v8nv8ck.1
Related research article	Edith Perret, Felix A. Reifler, Ali Gooneie, Rudolf Hufenus Tensile study of melt-spun poly(3-hydroxybutyrate) P3HB fibers: Reversible transformation of a highly oriented phase Polymer DOI: https://doi.org/10.1016/j.polymer.2019.121668

**Table:** 

**Value of the data** • The WAXD patterns highlight the highly-oriented non-crystalline mesophases in amorphous and semi-crystalline polymers. • The data shows the reversibility behaviour of the mesophase in P3HB under cyclic tensile loading. • This data can be compared to other WAXD and SAXS measurements from other P3HB fibers and films. • The data is of high interest to the field of bio-polymers and is potentially useful for the further development of melt-spinning of P3HB fibers. • The detailed description of WAXD and anisotropic 2D SAXS pattern data analysis of melt-spun fibers is potentially useful to many researchers.

## 1 Data

### 1.1 Equatorial reflections in WAXD patterns of amorphous fibers

**Fig. 1:**
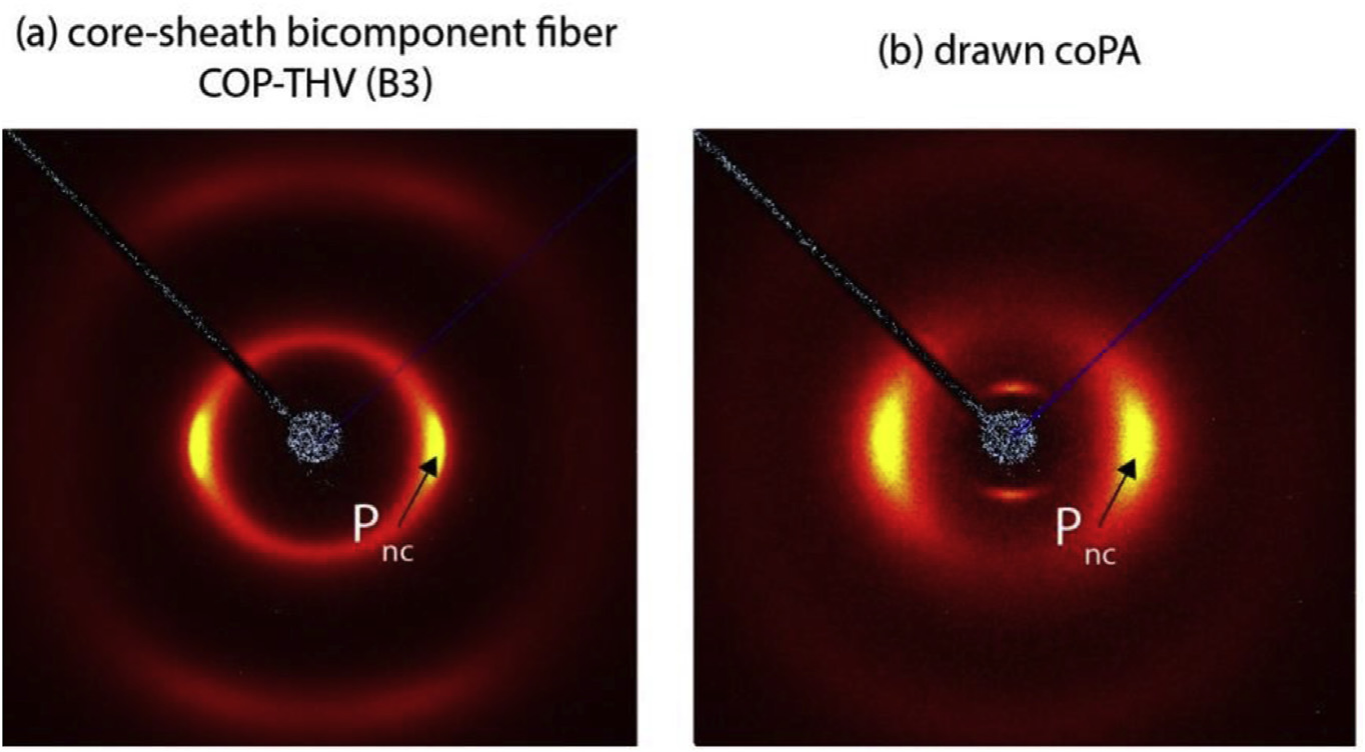
WAXD patterns of (a) a bicomponent fiber of a cyclo-olefin polymer (COP) in the core and a tetrafluoroethylene-hexafluoropropylene-vinylidene fluoride terpolymer (THV) in the sheath (draw ratio 2.5), and (b) a copolyamide (coPA) fiber [2], [3].

Wide-angle x-ray diffraction (WAXD) patterns of a bicomponent fiber of a cyclo-olefin polymer (COP) in the core and a tetrafluoroethylene-hexafluoropropylene-vinylidene fluoride terpolymer (THV) in the sheath (draw ratio 2.5), and a copolyamide (coPA) fiber [2], [3] are shown in (Fig. 1).

### 1.2 Molecular dynamics simulations and scattered intensity calculations of P3HB

Detailed explanations about the molecular dynamics simulations and scattered intensity calculations can be found in the article by Perret et al. [1].

Table 1 summarizes the calculated backbone torsional angles of a P3HB chain for different stretch factors. Each angle was averaged over all dihedrals along the chain over time during the molecular dynamics equilibration run. The deviations of the torsion angles from the perfect planar zigzag conformation (180°) are given in parentheses. Previously published values from Orts et al. and Tanaka et al. are also given in Table 1.

**Table 1:** Backbone torsional angles (all values are in degrees) of the simulated P3HB chains with various stretch factors.

Torsions (same notation as Orts et al. [4])	Stretch factors			Orts et al. [4]	Tanaka et al. [5] B
	1.54	1.7	2		
C3–O1–C1–C2	117.6 (−62.4)	120.1 (−59.9)	119.1 (−60.9)	112.6 (−67.4)	155.5 (−24.5)
O1–C1–C2–C3	−153.2 (26.8)	−154.4 (25.6)	−161.0 (19.0)	−168.9 (11.1)	167.9 (−12.1)
C1–C2–C3–O1	−124.3 (55.7)	−131.7 (48.3)	−153.4 (26.6)	−113.2 (66.8)	135.7 (−44.3)
C2–C3–O1–C1	170.0 (−10.0)	169.2 (−10.8)	167.6 (−12.4)	172.1 (−7.9)	−178.5 (1.5)

**Fig. 2:**
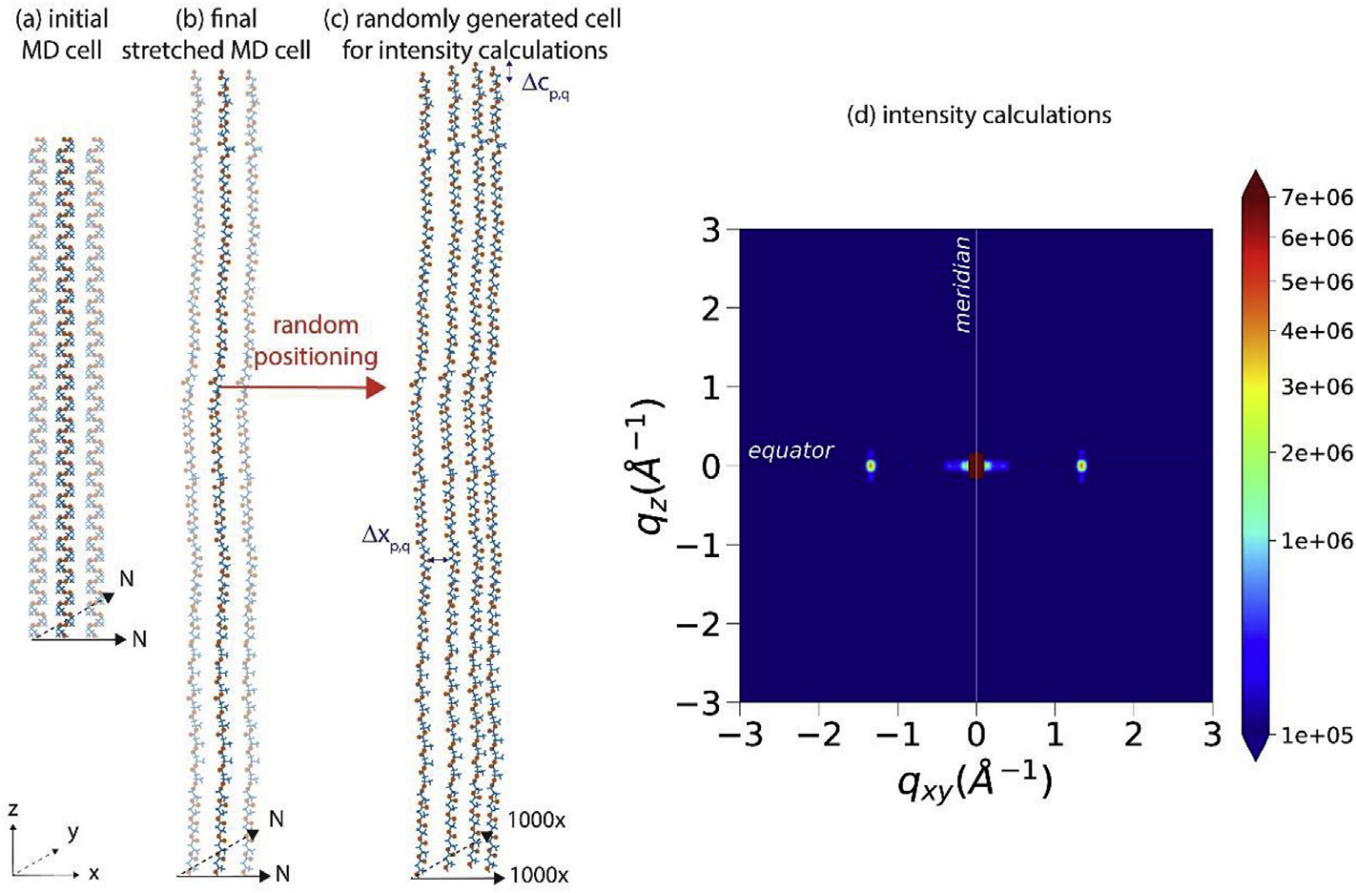
Schematic representation of molecular dynamics simulations of the stretching of a cell of helical chains (a-b) and (c) a cell of randomly positioned 1000 × 1000 chains of the same conformation for calculations of the scattered intensity. (d) Scattered intensity with random slippage of −3 Å to +3 Å.

We have calculated the scattered intensity from a box of 1000 × 1000 stretched, oriented and identical P3HB chains. These chains are irregularly distributed on the *xy* plane. The relative positions of the atoms within one single chain were extracted from the molecular dynamics simulations for a stretch factor of 1.7 after equilibration. A sketch of the simulation procedure is given in Fig. 2a–c. The scattered intensity calculation with random slippage of −3 Å to + 3 Å along the chain axis is shown in Fig. 2d.

### 1.3 Azimuthal profiles of WAXD patterns from P3HB fibers

#### 1.3.1 P_nc_ reflection

**Fig. 3:**
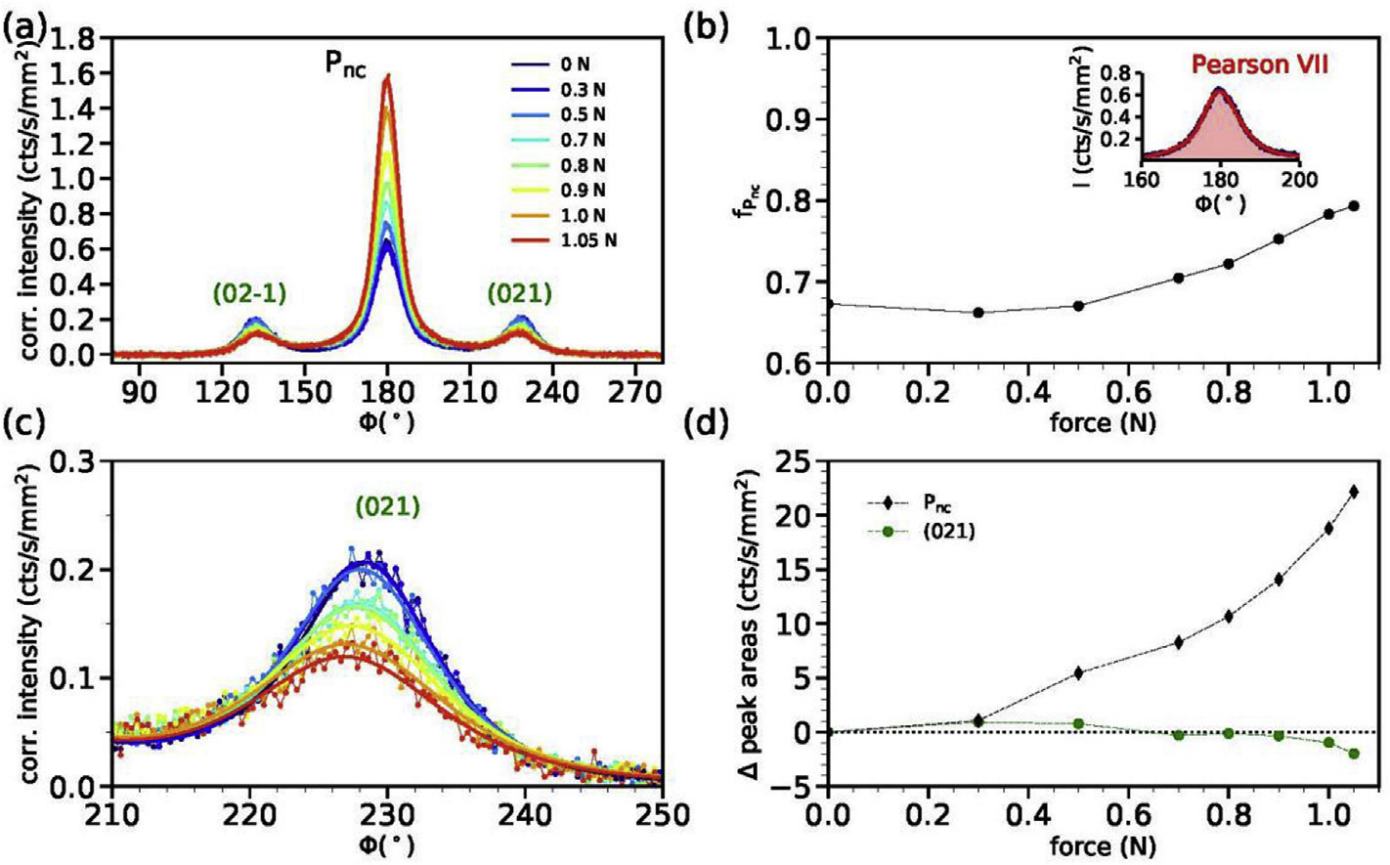
(a) Azimuthal profiles of the P_nc_/(021) annulus of fiber (I). (b) Orientation factors of the P_nc_ peak as a function of applied force. The inset shows a fitted Pearson VII curve to the P_nc_ peak at zero force. (c) Close-up of the (021) peak intensity. (d) Changes in peak intensities with respect to the initial peak intensity for zero force, Δ peak area (cts/s/mm^2^) = area-area(0N). The changes in the P_nc_ peak area at ϕ = 180° are shown as black diamonds and changes in the (021) peak area as green circles.

The azimuthal profile across the P_nc_ reflection is shown in Fig. 3a and the orientation factor in Fig. 3b. The (021) intensity is shown in Fig. 3c. Fig. 3d summarizes the changes in the intensities of peak areas with respect to the intensity measured at zero force for the P_nc_ (black diamonds) and the (021) reflection (green circles).

#### 1.3.2 Reflection (020)

**Fig. 4:**
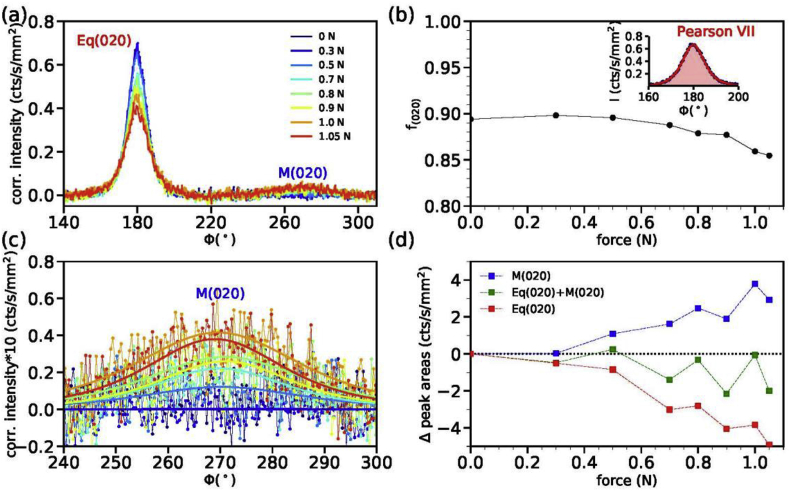
(a) Azimuthal profiles of the (020) annulus of fiber (I) for various applied forces. (b) Orientation factors of the (020) planes as a function of the applied force. The inset shows a fitted Pearson VII curve to the Eq(020) peak. (c) Close-up of the peak intensity of M(020), measured around ϕ = 270°. (d) Changes in peak intensities with respect to the initial peak intensity for zero force, Δ peak area (cts/s/mm^2^) = area-area(0N). The changes in the meridional peak area, M(020), are shown as blue squares, the changes in the equatorial peak area at ϕ = 180°, Eq(020), are shown as red squares; and the sum of the changes of both peaks as green squares.

**Fig. 5:**
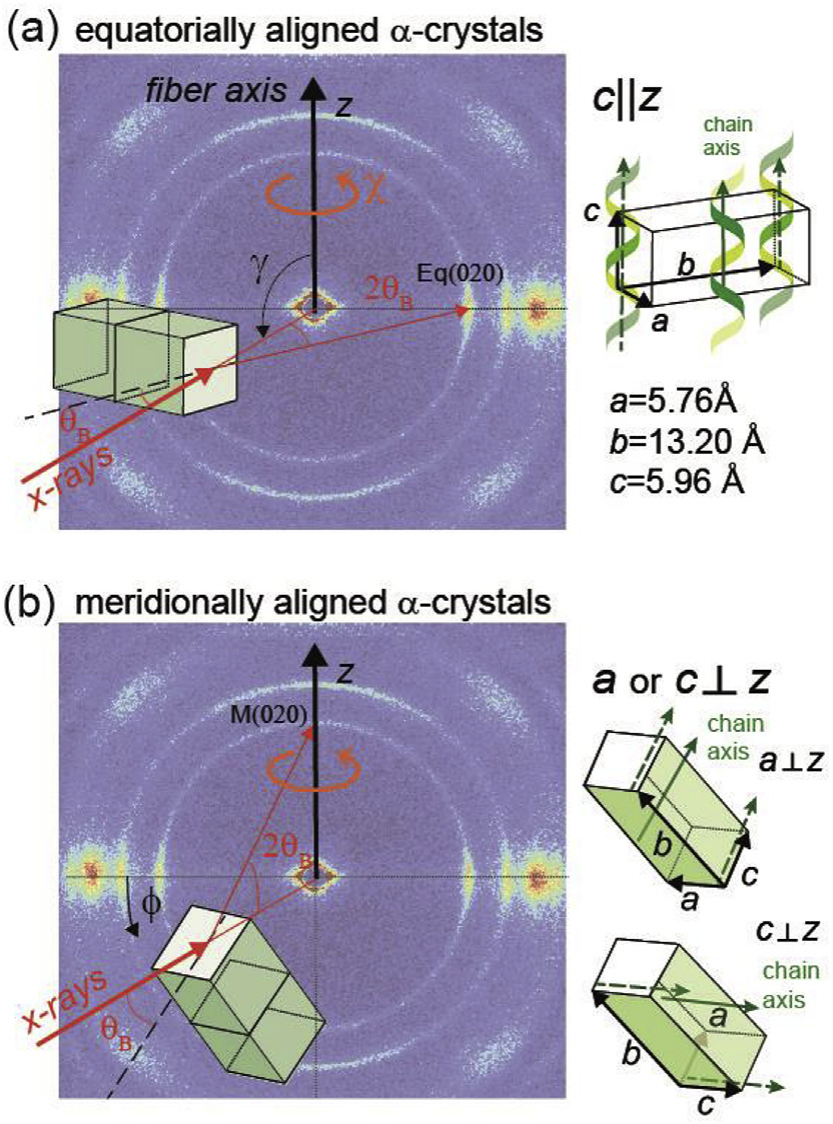
(a) Schematic of equatorially aligned α-form crystals. The molecular chain axis is parallel to the fiber axis. A sketch of the orthorhombic unit cell is given on the right. (b) Schematic of transversally grown (meridionally aligned) α-form crystals. Here, the short axis *a* or *c* is perpendicular to the fiber axis, whereas the long axis *b* is slightly tilted away from the fiber axis. Meridional reflections M(hk0) are observed when the corresponding planes (hk0) are tilted by the Bragg-angle, Θ_B_, away from the incident x-rays and either *a* or *c* is perpendicular to the fiber axis. All α-form crystals have a rotational symmetry around the fiber axis, *z* (rotation around χ). Some α-form crystals are randomly oriented with rotations in ϕ or γ, leading to the observed rings in the WAXD patterns.

Azimuthal profiles are shown in Fig. 4a for fiber (I) and were extracted from the WAXD patterns by summing the (020) annulus. The orientation parameters, f_(020)_ are shown in Fig. 4b for the equatorial reflection, Eq(020), which is located at 180° of the azimuth. From Fig. 4a it is seen that a broad reflection, labeled as M(020), appears along the meridional direction at ϕ = 270° for high tensions. A close-up of the latter reflection is shown in Fig. 4c. The Eq(020) and M(020) peak were fit to Pearson VII functions and the corresponding peak areas were integrated (inset Fig. 4b). The changes in the peak areas as a function of applied force with respect to the initial peak area measured at zero force are plotted in Fig. 4d. The specific orientation of crystallites that cause the equatorial and meridional reflections are illustrated in Fig. 5.

### 1.4 Positions of equatorial α-form reflections of P3HB fibers

Table 2 summarizes the fitted peak positions of the equatorial (020) and (110) reflections of fiber (I).

**Table 2:** Fiber (I) subjected to increasing forces on the tensile stage: resulting stresses, strains and impact on various features of the WAXD pattern. Forces exceeding 1.05 N resulted in breakage of the fiber.

Force (N)	Stress (MPa)	Strain (%)	Position of Eq(020) (°) ±0.06°	*d*_(020)_ (Å) ±0.005 Å	f _(020)_ (Fig. 4b)	Position of Eq(110) (°)±0.06°	*d*_(110)_ (Å)±0.005 Å
0	0	0	13.36	6.63	0.89	16.69	5.31
0.30	49	0.1	13.37	6.62	0.90	16.70	5.31
0.50	81	9.8	13.39	6.61	0.90	16.75	5.29
0.70	113	20.2	13.40	6.61	0.89	16.76	5.29
0.80	130	26.3	13.42	6.60	0.88	16.78	5.28
0.90	146	35.8	13.42	6.60	0.88	16.79	5.28
1.00	162	49.3	13.43	6.59	0.86	16.78	5.28
1.05	170	58.7	13.44	6.59	0.85	16.79	5.28

### 1.5 Structure reversibility under cyclic loading of P3HB fibers

**Fig. 6:**
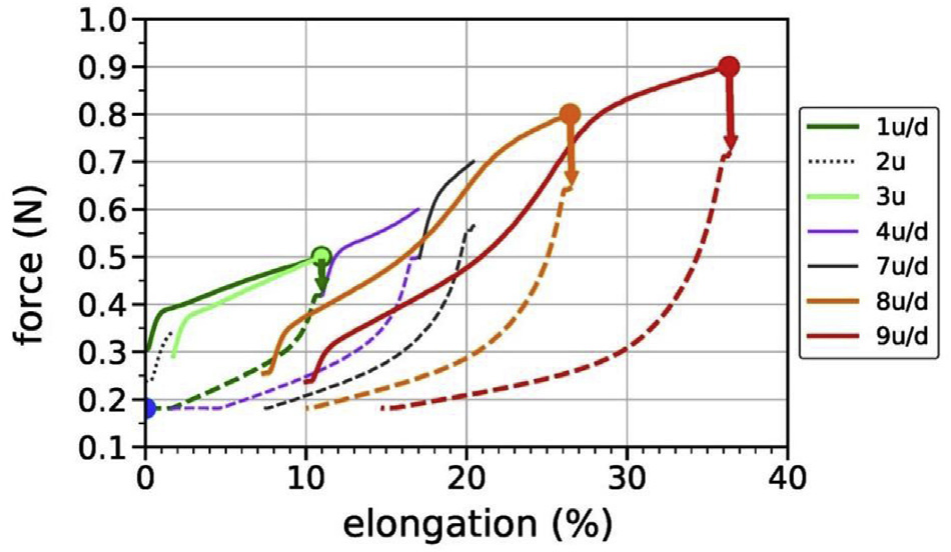
Load-elongation curves of fiber (I) for cyclic changes in tensile loading. The steps with increasing elongation ‘u’ are shown as plain colored lines, whereas dashed lines represent subsequent steps with decreasing elongation ‘d’. The gaps between consecutive steps are due to relaxation during WAXD data acquisition, indicated with arrows.

Load-elongation curves of various consecutive steps are shown in Fig. 6.

Table 3 provides additional data such as starting and end elongations/forces for each step as well as relaxation times between steps. The table lists all of the performed steps during the load-cycling tests. Two-theta curves shown in the article by Perret et al. [1] and azimuthal profiles were extracted from the WAXD patterns labeled as X0 (not shown in Table 3, starting WAXD pattern), X1, X2, X4, X10, X12, X13.

**Table 3:** Fiber sample (I): cyclic change of load on the tensile stage: details of forces, elongation and relaxation in the load-elongation curves for the various steps as presented in Fig. 6. Starting position 0.30 mm equates to 0.0% elongation. Bold letters indicate WAXD patterns that were analyzed in the article by Perret et al. [1].

label	direction of load change	starting force [N]	start [mm]	starting elongation [%]	end force [N]	end [mm]	end elongation [%]	relaxation time until next step [min]	WAXD pattern
**1u**	**up**	**0.30**	**0.30**	**0.0**	**0.50**	**1.78**	**11.0**	**46**	**X1**
**1d**	**down**	**0.42**	**1.79**	**11.0**	**0.18**	**0.30**	**0.0**	**146**	**X2**
2u	up	0.24	0.29	−0.1	0.34	0.53	1.7	41	X3
**3u**	**up**	**0.29**	**0.53**	**1.7**	**0.50**	**1.78**	**11.0**	**53**	**X4**
4u	up	0.42	1.79	11.0	0.60	2.59	17.0	37	X5
4d	down	0.50	2.60	17.0	0.18	0.48	1.3	8	–
5u	up	0.18	0.48	1.3	0.27	0.92	4.6	3′330^a^	X6.1^a^ X6.2^a^
6u	up	0.29	0.92	4.6	0.61	2.59	17.0	52	X7
7u	up	0.50	2.60	17.0	0.70	3.06	20.4	61	X8
7d	down	0.57	3.07	20.5	0.18	1.30	7.4	131	X9
**8u**	**up**	**0.25**	**1.29**	**7.3**	**0.80**	**3.87**	**26.4**	**41**	**X10**
8d	down	0.65	3.89	26.6	0.18	1.65	10.0	43	X11
**9u**	**up**	**0.24**	**1.65**	**10.0**	**0.90**	**5.21**	**36.4**	**43**	**X12**
**9d**	**down**	**0.72**	**5.22**	**36.4**	**0.18**	**2.28**	**14.7**	**–**	**X13**

a 3′330 min equates 55.5 h. WAXD pattern X6.1 was recorded at the beginning, X6.2 was recorded at the end of the relaxation time.

**Fig. 7:**
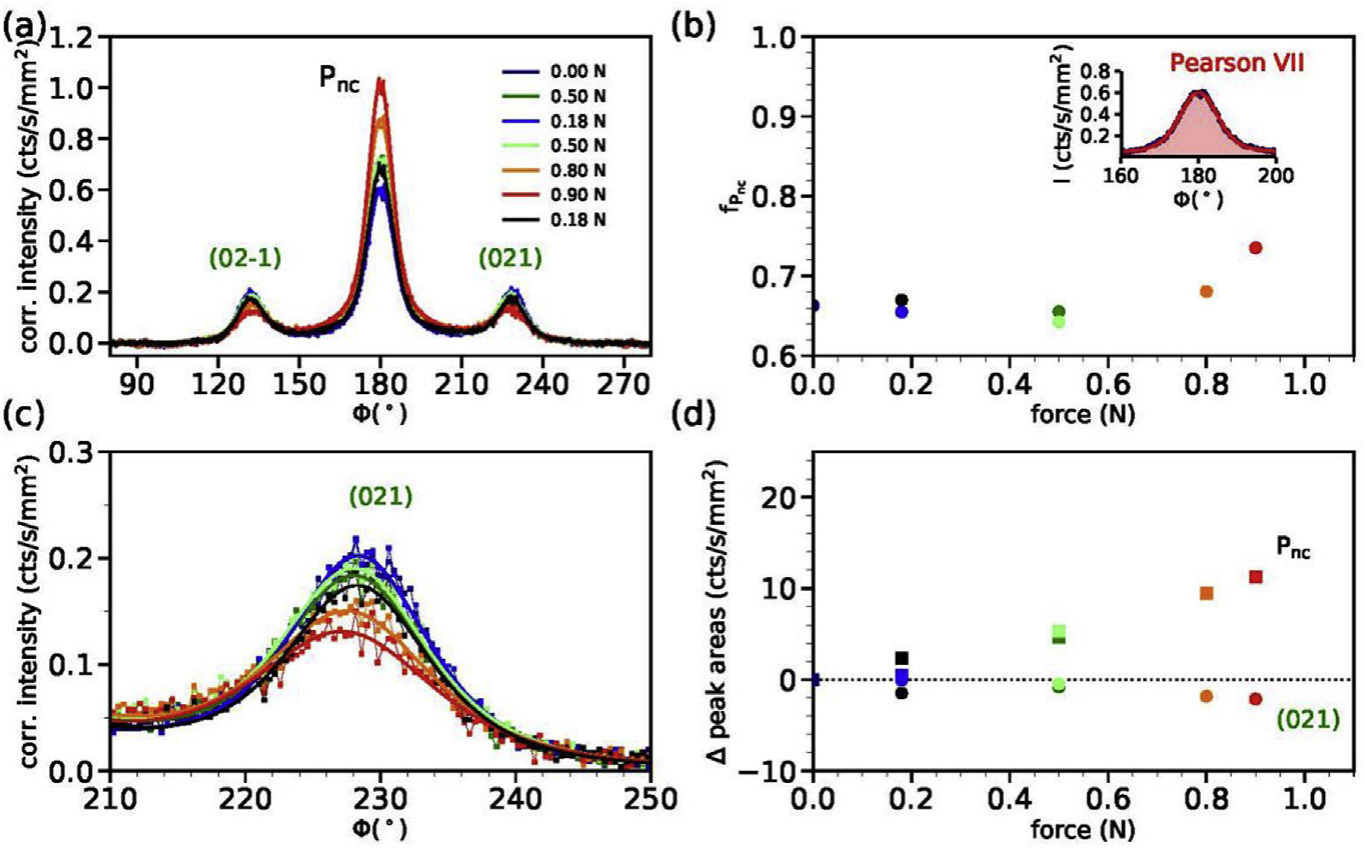
(a) Azimuthal profiles of the P_nc_/(021) annulus of fiber (I) for cyclic loading. (b) Orientation factors of the P_nc_ peak as a function of applied force. The inset shows a fitted Pearson VII curve to the P_nc_ peak at zero force. (c) Close-up of the (021) peak intensity. (d) Changes in peak intensities with respect to the initial peak intensity for zero force, Δ peak area (cts/s/mm^2^) = area-area(0N). The changes in the P_nc_ peak area at ϕ = 180° are shown as squares, changes in the (021) peak area as circles.

Fig. 7a shows azimuthal profiles across the P_nc_ reflection under cyclic loading and the orientation factor is shown in Fig. 7b. The intensity changes to the (021) peak are shown in Fig. 7c. Changes in the P_nc_ and (021) peak areas are shown in Fig. 7d.

### 1.6 SAXS data analysis of P3HB fibers

The results of *L* and *H* as a function of applied tension are summarized in Table 4 and Table 5.

**Table 4:** Structural parameters extracted from peak (1), which are attributed to PCL lamellae.

SAXS plot	force (N)	peak (1) position (°)	long spacing *L*_1_ (nm)	peak (1) mer. width (°)	coherence length *H*_1_ (nm)	peak (1) transversal width (°)	Lamellar diameter *D*_1_ (nm)
(a)	0.00	0.59	14.9	0.64	12.4	0.29	27.1
(b)	0.50	0.56	15.8	0.42	18.8	0.38	21.0
(c)	0.18	0.59	14.9	0.47	16.8	0.33	23.8
(d)	0.70	0.52	17.1	0.33	24.4	0.45	17.6
(e)	0.19	0.62	14.3	0.65	12.2	0.39	20.3
(f)	0.70	0.53	16.6	0.35	22.7	0.46	17.3
(g)	0.19	0.61	14.5	0.98	8.1	0.48	16.4

**Table 5:** Structural parameters extracted from peak (2), which are attributed to P3HB lamellae.

SAXS plot	force (N)	peak (2) position (°)	long spacing *L*_2_ (nm)	peak (2) mer. width (°)	coherence length *H*_2_ (nm)	peak (2) transversal width (°)	Lamellar diameter *D*_2_ (nm)
(a)	0.00	1.15	7.7	0.47	16.8	0.52	15.3
(b)	0.50	1.00	8.8	0.51	15.7	0.51	15.5
(c)	0.18	1.08	8.2	0.56	14.2	0.52	15.2
(d)	0.70	0.92	9.6	0.53	14.9	0.55	14.5
(e)	0.19	1.11	8.0	0.45	17.5	0.52	15.3
(f)	0.70	0.94	9.4	0.52	15.4	0.55	14.3
(g)	0.19	1.06	8.3	0.38	20.6	0.53	14.9

**Fig. 8:**
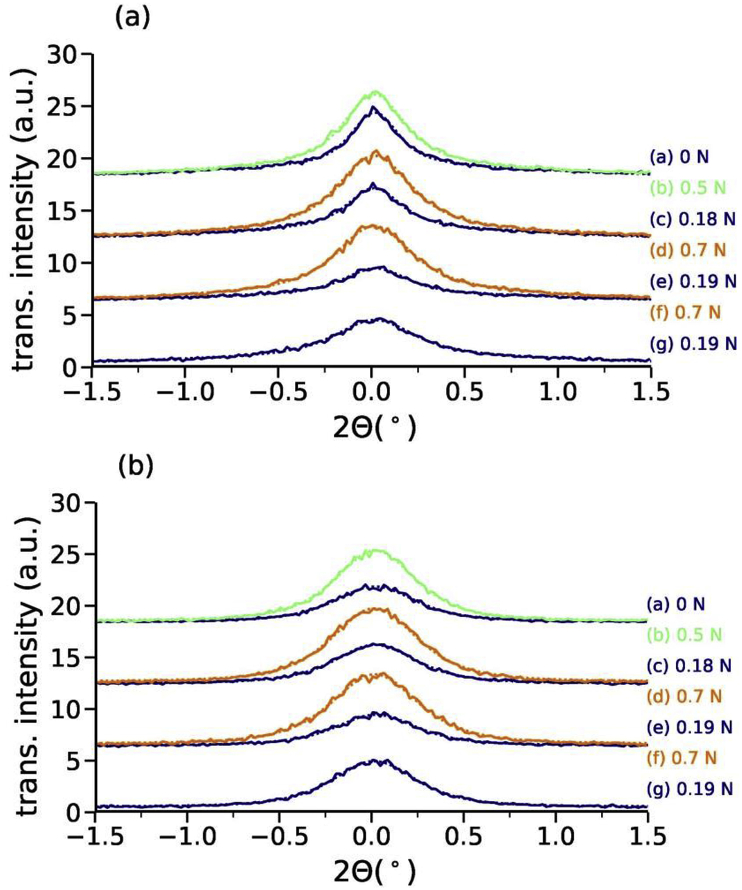
Transversal profiles (a) across peak (1) and (b) across peak (2). The curves are offset for better visibility.

Lamellar diameters are extracted from the SAXS patterns by fitting the averaged intensity of the transversal areas to a Pearson VII function. The transversal intensity distribution is shown in Fig. 8 for the first and second peak. The background in the transversal scans across peak (1) was fit with a broad Pearson VII function and across peak (2) with a linear background.

## 2 Experimental design, materials, and methods

### 2.1 WAXD on coPA and bicomponent core/sheath COP/THV fibers

Fiber bundles were mounted on a custom-made sample holder. For the drawn COP and CoPA fibers, the fiber bundles consisted of 8 and 16 single filaments, respectively. WAXD patterns were recorded on an Xcalibur PX four-circle single-crystal diffractometer (Oxford Diffraction, Yarnton, Oxfordshire, UK; κ geometry; Mo Kα1 radiation, λ = 0.709 26 Å, CCD area detection system). CoPA have been processed by high-speed melt spinning and drawing to produce monofilaments with linear mass densities of 6.5 and 3.85 tex (mgm^−1^), respectively. More information can be found in the articles by Leal and Reifler et al. [2], [3].

### 2.2 Molecular dynamics simulations

The molecular dynamics simulation and scattered intensity calculations have been previously described in detail in the article by Perret et al. [1]. Part of the text below has been taken from the said article and is therefore put into quotes.

"Molecular dynamics simulations were carried out to elucidate the changes in the conformation of a P3HB chain during a uniaxial stretching parallel to the chain axis. The initial conformation of the P3HB chain was taken from the helical structures in the α-form crystal unit cell previously published by Wang et al. [6]. The non-bonded and bonded interactions were defined according to the recent implementation of the General AMBER Force Field (GAFF) used for P3HB [7]. This description of the P3HB chain in GAFF combines the standard 12-6 Lennard-Jones potential and electrostatic interactions for non-bonded interactions, with bond, angle, and dihedral potentials for bonded interactions [8], [9]. The details are given by Glova et al. [7]. Our starting orthogonal simulation box contained a helical α-P3HB chain with 46 monomers and was deformed parallel to the chain axis until its dimension was scaled by a predefined stretch factor. This factor was set to 1.54, 1.7, and 2 in different simulations. After the deformation step, the atoms with the maximum and minimum positions along the chain axis were frozen in space and the rest of the atoms were free to equilibrate. The simulation time step was 0.5 fs with the overall run time of 1 and 1.5 ns for the deformation and equilibrium steps, respectively. The temperature was fixed during the entire simulation at 298 K.

We have performed scattered intensity calculations in order to show that an irregular non-crystalline structure of highly-oriented and stretched P3HB chains leads to similar scattered intensity patterns as the observed ones. Specifically, we have calculated the scattered intensity from a box of 1000 × 1000 stretched, oriented and identical chains. These chains are irregularly distributed on the *xy* plane. The relative positions of the atoms within one single chain were extracted from the molecular dynamics simulations for a stretch factor of 1.7 after equilibration. A sketch of the simulation procedure is given in Fig. 2a–c."

### 2.3 P3HB fibers

The materials and experimental methods for in-situ tensile tests on P3HB fibers have been previously described in detail in the article by Perret et al. [1]. Part of the text below has been taken from the said article and is therefore put into quotes.

"Two fiber samples (I,II) were melt-spun from modified P3HB provided by Biomer (Krailling, Germany) on a customized pilot melt spinning plant originally built by Fourné Polymertechnik (Alfter-Impekoven, Germany).

WAXD patterns of two fibers and SAXS patterns fiber (I) were recorded on a Bruker Nanostar U diffractometer (Bruker AXS, Karlsruhe, Germany) with a beam defining pinhole of 300 μm, with Cu Kα radiation (λ = 1.5419 Å) and a VÅNTEC-2000 MikroGap area detector. The tensile stress was applied using a TS 600 tensile stage (Anton Paar GmbH, Graz, Austria) equipped with load cell LC-5N. The calculation of applied tensile stresses is given below in subsection 2.3.1. Single filaments were used for all WAXD and SAXS measurements, which were performed in two separate experiments with distances of 16.8 cm and 144.4 cm, respectively, between the sample and the active detector area. The filaments were glued on top of supports and held by tensile stage grips. The filaments were elongated stepwise at an elongation rate of 0.1 mm/min. During elongation, the measured force increased, and the elongation step was automatically stopped when the pre-set nominal force value for the respective step was reached. Immediately after stopping, a WAXD pattern was recorded for 30 minutes or a SAXS pattern was recorded for 2 h, respectively. During data collection, the grip position was kept constant by the tensile stage mechanism, while the filament underwent some relaxation. This relaxation resulted in a lower starting force for the subsequent step.

The recorded WAXD/SAXS patterns were analyzed with the evaluation software DIFFRAC.EVA (version 4.2., Bruker AXS, Karlsruhe, Germany) and python codes. Real space *d*-spacings between planes corresponding to 2Θ_B_ values of the respective (hkl) reflections were calculated applying Bragg's law [10]. Peaks in, e.g., azimuthal WAXD scans were fit with Pearson VII distribution functions using python codes [11]. Herman's equation (subsection 2.3.2, Eq. 2) was applied in order to extract the orientation parameter f_(hk0)_ of the α-form crystals [12]. If ${\text{f}}_{\left(\text{hk}0\right)}=1$ then the (hk0) planes of the crystals are completely aligned parallel to the fiber axis and if ${\text{f}}_{\left(\text{hk}0\right)}=0$, then the crystals are randomly oriented. Long-spacings, coherence lengths and lamellar sizes were calculated by analyzing meridional and transversal areas of the SAXS pattern (Eq. 9 and Eq. 10). The performed intensity corrections to the WAXD/SAXS patterns are explained in subsections 2.3.3-2.3.6."

#### 2.3.1 Tensile stress calculations

The tensile stress corresponding to the measured forces was calculated from the linear mass density, LMD, of the fibers with LMD=m/L=V∗ρ/L, hence V/L=LMD/ρ, where m is the mass, L the length and ρ the density (1.2 g/cm^3^ was chosen for all samples). With S=F/A=F∗L/V where S is the stress, F the force and A=V/L the cross sectional area, it follows:

(Eq. 1) $$S=F\frac{\rho }{LMD}$$

#### 2.3.2 Crystal orientation: Herman's equation

The crystal orientation is defined using Herman's equation [12], which was previously generalized for a set of three crystallographic axes by Stein [13]. The crystalline orientation is defined as

(Eq. 2) $${\text{f}}_{\left(\text{hkl}\right)}=\frac{3\langle {\text{cos}}^{2}\phi -1\rangle }{2}$$

where $\cos^{2}\;\phi$ reflects the azimuthal spread of the (hkl) reflection. Here, the azimuthal angle is zero at the maximum of the reflection. Assuming rotational symmetry around the fiber axis, the term $\cos^{2}\;\phi$ is given by

(Eq. 3) $$\langle \cos^{2}\;\phi \rangle =\frac{\int_{0}^{\pi /2}I\left(\phi \right)\cos^{2}\;\phi \;\sin\;\phi \;\text{d}\phi }{\int_{0}^{\pi /2}I\left(\phi \right)\sin\;\phi \;\text{d}\phi }$$

where I(ϕ) is the intensity diffracted from the (hkl) planes. The following holds for equatorial (hk0) reflections: If ${\text{f}}_{\left(\text{hk}0\right)}=1$ then the (hk0) planes of the crystals are completely aligned parallel to the fiber axis. If ${\text{f}}_{\left(\text{hk}0\right)}=0$, then the crystals are randomly oriented. Note that we have used the same equation to calculate an orientation factor for the P_nc_ phase, which is thought to be non-crystalline.

#### 2.3.3 Intensity corrections: thinning of the fiber

When applying a tensile stress to a fiber, the fiber stretches and gets thinner due to the Poisson effect. Therefore, the x-ray beam hits less material and the overall diffracted intensity decreases. To correct for this intensity loss we have assumed a Poisson's ratio of 0.5, meaning that the volume of the fiber stays constant upon stretching. A specific tensile force has been applied to an initial fiber section of volume *V*_1_ with a cross-section area of *A*_1_ and a known length *l*_1_ (measured length between grips of the tensile stage at zero force) resulting in a stretching of the fiber to a final length *l*_2_ (measured).

(Eq. 4) $$V_{1}=l_{1}A_{1}=l_{2}A_{2}$$

The measured intensities, *I*_meas_, in the WAXD patterns at a certain force have therefore been multiplied with a correction factor, *C*, which was calculated from the measured elongations after each tensile stretch.

(Eq. 5) $${\text{I}}_{\text{corr}}=\text{C}\times {\text{I}}_{\text{meas}}$$

(Eq. 6) $$\text{C}=\frac{l_{2}}{l_{1}}$$

#### 2.3.4 Intensity corrections: Lorentz-polarization correction

When X-rays are diffracted by a lattice plane they are partially polarized, which leads to an intensity reduction that can be expressed as a function of the diffraction angle. For a completely unpolarized primary beam the measured intensity is divided by the polarization factor:

(Eq. 7) $$P=\frac{1}{2}\left(1+cos^{2}\left(2\theta \right)\right)$$

Constructive interference also takes place in the vicinity of the Bragg angle since X-rays are not strictly monochromatic. A detector of finite aperture can count more photons at low (or equally at high) angle, and less when the scattering angle approaches 90°. The amount of the Debye-Scherrer cone measured is simply inversely proportional to the sine of the scattering angle 2θ, i.e. 1/sin(2θ). Additionally, the probability of the crystallites to have the planes in the correct orientation for Bragg scattering is inversely proportional to the sine of the incident Bragg angle θ and φ_hkl_, with the latter being the angle between the plane normal and the fiber axis. To account for these effects the measured intensity is divided by the Lorentz factor [14], [15]:

(Eq. 8) $$L=\frac{1}{\sin\;2\;\theta \;\sin\;\theta \;\sin\varphi_{hkl}}=\frac{1}{2{\text{sin}}^{2}\theta \;\cos\;\theta \;\sin\varphi_{hkl}}$$

#### 2.3.5 Intensity corrections: equatorial/meridional profiles

The equatorial profiles were extracted from the evaluation software DIFFRAC.EVA (version 4.2., Bruker AXS, Karlsruhe, Germany). The intensity in the equatorial/meridional sector was summed with the ‘algorithm of frame integration’ and was divided by the acquisition time (1800s) and normalized to an area of 1mm^2^. Subsequently, the profiles were corrected for the thinning of the fibers.

#### 2.3.6 Intensity corrections: azimuthal profiles

The azimuthal profiles were extracted from the evaluation software DIFFRAC.EVA (version 4.2., Bruker AXS, Karlsruhe, Germany). The intensity in the annulus was summed with the ‘algorithm of frame integration’ and was divided by the acquisition time (1800s) and normalized to an area of 1mm^2^ by the software. Further corrections to the profiles were performed with python codes: First the azimuthal profiles of the (020) annulus were corrected for the background arising from the air/diffuse scattering and amorphous phase by subtracting a normalized azimuthal profile from an adjacent annulus located between 11 and 12.2° in 2Θ. An equatorial streak makes this subtraction necessary. This correction was not performed for the P_nc_/(021) annulus. Subsequently, the profiles were corrected for the thinning of the fiber. Additionally, the profiles have been corrected for slight dips in intensity at ϕ = 0 and ϕ = 180°, which were caused by Kapton wires that held the beamstop. For this purpose, the 360° profiles were mirrored at 90° and the maximum values of the overlapped intensities in the peak area (around 180°) were taken. Symmetrized and averaged profiles about the equator were subsequently background corrected (arising from randomly oriented crystals or remaining amorphous phase). For the background, the intensities in a specific region on the azimuth were averaged, where a flat uniform background was observed (300–305° for (020), 260–280° for P_nc_ sector). The data was typically binned with 8 or 4 bins.

#### 2.3.7 SAXS patterns: extraction of structural parameters

The lamellar long spacings, *L*, can be extracted from the meridional peak positions (1) and (2) using the expression

(Eq. 9) $$\text{L}=\frac{2\text{π}}{{\text{q}}_{\text{LM}}}=\frac{\text{λ}}{\left(2\;\sin{\text{θ}}_{\text{LM}}\right)}$$

where $q_{LM}=\frac{4\pi }{\lambda }\sin\theta_{LM}$ is the scattering vector and $\theta_{LM}$is half the scattering angle at the lamellar reflection and λ is the wavelength.

The coherence length *H* along the fiber direction and lamellar stack diameters *D* perpendicular to the fiber direction are calculated from the width of the lamellar reflections along the meridian and the width of the reflections in transversal scans using the Scherrer equation [16].

(Eq. 10) $$size=\frac{0.9\lambda }{\Delta \left(2\theta \right)\cos\;\theta }\approx \frac{0.9\lambda F}{\sqrt{FWHM^{2}-b^{2}}}$$

where *size* stands for either the coherence length H or the lamellar diameter D, F is the fiber-to-detector distance, FWHM is the full width at half-maximum of the reflection and b is the instrumental broadening which is negligibly small (b≈0). The equation makes use of small-angle approximations, cosθ≈1.
